# Influence of type of cooking fuel on risk of hypertension among reproductive-age women in sub-Saharan Africa: insights from nationally representative cross-sectional surveys

**DOI:** 10.1093/inthealth/ihad060

**Published:** 2023-08-10

**Authors:** Castro Ayebeng, Joshua Okyere, Kwamena Sekyi Dickson

**Affiliations:** Department of Population and Health, University of Cape Coast, Cape Coast, Ghana; Department of Population and Health, University of Cape Coast, Cape Coast, Ghana; Department of Nursing, College of Health Sciences, Kwame Nkrumah University of Science and Technology, Kumasi, Ghana; Department of Population and Health, University of Cape Coast, Cape Coast, Ghana

**Keywords:** cooking fuel, Demographic and Health Survey, hypertension, public health

## Abstract

**Background:**

Nearly one-third of the world's population (2.4 billion people) rely on unclean cooking fuel sources. The study assessed the association of the type of cooking fuel and hypertension risk in sub-Saharan Africa (SSA).

**Methods:**

The study analysed pooled data from 97 942 individuals in the Demographic and Health Survey (DHS) between 2014 and 2021 in 10 SSA countries. Univariate, bivariate and multivariate analyses were performed, including basic descriptive statistics and binary logistic regression. The independent variable of interest was the type of cooking fuel, while hypertension served as the outcome variable.

**Results:**

Women using unclean cooking fuel were 1.21 times more likely to be hypertensive compared with those using clean cooking fuel (adjusted odds ratio [aOR] 1.21 [95% confidence interval {CI} 1.11 to 1.31]). Older age (aOR 5.78 [95% CI 5.04 to 6.62]), higher education (aOR 1.14 [95% CI 1.05 to 1.23]), being married (aOR 1.64 [95% CI 1.49 to 1.80]), working in sales and services occupations (aOR 1.34 [95% CI 1.24 to 1.44]), frequent health facility visits (aOR 1.59 [95% CI 1.51 to 1.68]), higher wealth index and exposure to media were significantly associated with hypertension risk.

**Conclusions:**

Efforts to reduce reliance on unclean cooking fuel at both the household and population levels need to be intensified in SSA countries. Promoting the use of clean cooking technologies and fuels and implementing supportive policies for transitioning from unclean cooking fuels are crucial. Targeted interventions to reduce hypertension risk in SSA should focus on women using unclean cooking fuel, older women, individuals from wealthier households and those with higher education levels.

## Background

Hypertension remains a serious public health concern. The World Health Organization (WHO) estimates that nearly 1.28 billion adults live with this condition, with 46% of this population being unaware of their hypertensive status.^1^ The burden of hypertension is projected to reach 1.56 billion by 2025.^2^ As a notable risk factor for cardiovascular diseases, hypertension claims 9.4 million lives each year.^3^ This implies that reducing the risk of hypertension would contribute significantly to reducing cardiovascular diseases and improving quality of life, thus emphasising a need to identify modifiable factors that support lowering hypertension risk among the population.

There is a preponderance of literature that has found consistently significant risk factors for hypertension, including older age,^4^ being overweight and obese,^5^ smoking,^6^ a sedentary lifestyle^7^ and high sodium intake.^8^ In addition to these risk factors, air pollution has been found to significantly increase the risk of hypertension.^9,10^ The strong relationship between air pollution and hypertension risk is exacerbated by increasing exposure to industrial emissions, vehicular sources and indoor/household sources. However, in recent times there has been increasing scholarly interest in the latter, specifically the role of household cooking fuels as a risk factor for hypertension.

Nearly one-third of the world's population (2.4 billion people) rely on unclean cooking fuel sources (charcoal, firewood, grass/straw, dung, shrubs and agricultural crop waste),^11^ thus making unclean cooking fuel a major environmental risk factor for several adverse health outcomes, including hypertension. Nevertheless, there is a high level of inconsistency in the extant literature that has assessed the association between the type of cooking fuel and hypertension risk. While some studies have found a significantly positive association between the type of cooking fuel and the risk of hypertension,^12,13^ others have reported no association^14,15^ or significant inverse associations.^16,17^ These inconsistencies in the current knowledge of the subject necessitate a need for more studies to shape the discourse.

Despite the many studies on the subject, the majority have been conducted in China.^12–14^ To the best of our knowledge, after an extensive literature search, no present study has investigated the association between the type of cooking fuel and hypertension risk in sub-Saharan Africa (SSA). The existing studies in SSA have examined the type of cooking fuel and general health outcomes, including adverse pregnancy outcomes,^18^ child nutritional status^19^ and respiratory illnesses.^20^ This presents a significant knowledge gap in understanding the extent to which the type of cooking fuel determines hypertension risk in SSA. The study tests the hypothesis that the type of household cooking fuel is significantly associated with the likelihood of being hypertensive. Hence, the aim of the study was to assess the association of the type of cooking fuel and hypertension risk in SSA using a nationally representative dataset.

## Materials

### Data

The study analysed data from the most recent Demographic and Health Surveys (DHS) (2014–2021) of 10 SSA countries (Benin, Burundi, Cameroon, Ghana, Gambia, Kenya, Lesotho, Madagascar, Mauritania and Zambia) that had the variables of interest included in the study. The DHS uses a cross-sectional study design and a two-stage stratified cluster sampling method to select nationally representative samples of women of reproductive age (15–49 y). The DHS is suitable for our study because it gathers comprehensive information on a variety of issues, including our variables of interest (hypertension status, type of cooking fuel) and other important demographic characteristics. The quality of the DHS data for such regional-level analysis is widely recognised and has consequently been used for numerous studies.^21–24^ A total sample of 150 254 women 15–49 y of age was drawn from the 10 countries; however, a weighted sample of 97 942 was used in the analyses after dropping all missing variables, to ensure the same sample across variables. Ethical approval for the use of the dataset was not required since it was drawn from a secondary data source. However, permission for its use was secured from Measure DHS.

### Study variables and measurements

#### Outcome variable

The study sought to examine the influence of the type of cooking fuel on the risk of developing hypertension. This study used hypertension as the outcome variable of interest. This was derived from the question ‘Have you ever been diagnosed with hypertension/high blood pressure?’. The response was captured as no=0 and yes=1.

#### Explanatory variable

The study used type of cooking fuel as the main independent variable. This was derived from the question ‘What type of fuel does your household mainly use for cooking?’. The response options included electricity, liquefied petroleum gas (LPG), natural gas, biogas, kerosine, coal lignite, charcoal, wood, straw/shrubs/grass, agricultural crops, animal dung or no food cooked in the household. Based on a standardised categorization of the type of cooking fuel,^22,23^ we grouped electricity, LPG, natural gas and biogas as clean cooking fuel (=0) and the other types of cooking fuel as unclean cooking fuel (=1). Similar categorization has been used in other studies.^19^ It should be noted that respondents who did not cook in the household were dropped from the analysis. Based on existing evidence, 10 independent variables known to have influence on the outcome^24–29^ were identified and accounted for in the analysis. These include the women's age (15–19=1, 20–24=2, 25–29=3, 30–34=4, 35–39=5, 40–44=6, 45–49=7); level of education (no education=0, primary=1, secondary and above=2), marital status (never married=1, married=2, formerly married=3), wealth index (poorest=1, poorer=2, middle=3, richer=4, richest=5), place of residence (urban=0, rural=1), visited a health facility in the last 12 months (yes=0, no=1), occupation (not working=0, professional/managerial/clerical=1, sales/service=2, agriculture=2, other=3), frequency of reading a newspaper or magazine (not at all=0, less than once a week=1, at least once a week=2, almost every day=3), frequency of listening to the radio (not at all=0, less than once a week=1, at least once a week=2, almost every day=3), and frequency of watching television (not at all=0, less than once a week=1, at least once a week=2, almost every day=3) (see Table 2).

### Analytical procedure

The analysis was conducted at two main levels using both descriptive and inferential analyses. The descriptive analysis involved the proportion of the respondents who were hypertensive in the selected countries. It also showed the sample and the proportions of the background characteristics by the outcome variable (hypertension). A χ^2^ test was used to examine the statistically significant difference between each explanatory variable and the outcome. Additionally, a binary logistic regression model was subsequently used in both bivariate and multivariate analyses to ascertain the significant association between the main independent variable (type of cooking fuel) and hypertension. This model was utilized due to the dichotomous nature of the outcome variable. In all, three models were fitted. In the first model we looked at the association between the type of cooking fuel and hypertension among the sampled population. In the second model we adjusted for the influence of other important independent variables such as age, level of education, marital status, wealth index, residence, occupation and exposure to media (newspaper/magazine, radio and television) in order to measure the true effect of the main independent variable on the outcome.

In the final model (model 3), together with other independent variables, we adjusted for the country variable to control for the effect of country variation and different time points for the data collection. However, before conducting the logistic regression analysis, a multicollinearity test was performed on each variable and the results showed that the variables in the models had a mean variance inflation factor (VIF) of 4.84, which shows the non-existence of multicollinearity. Using a 95% confidence interval (CI), the results were presented as adjusted odds ratios (aORs). The data were analysed using Stata version 14 (StataCorp, College Station, TX, USA). All estimates were sample weighted to address any sampling bias due to under- or oversampling of participants from the total population. This was attained by using the individual sampling weight variable, v005 in the dataset of the female's file. The survey command in Stata was used to adjust for the complex sampling structure of the data in the regression analysis. The dataset is freely accessible for download at https://dhsprogram.com/data/available-datasets.cfm.

## Results

### Distribution of hypertension status by country of residence

Table 1 shows the prevalence distribution of hypertension in the sampled population by country of residence in the selected countries and the corresponding survey year. The results revealed that the prevalence of hypertension (8.40%) among reproductive-age women was statistically significant (p<0.001) regarding the country of residence. Specific to country of residence, we observed the highest percentage of hypertensive women in Lesotho (17.57%), followed by Gambia (14.32%) and Mauritania (14.19%). In contrast, Burundi has the lowest percentage, with only 1.00% of women being hypertensive, followed by Cameroon (6.37%).

**Table 1.** Country, survey years and hypertension status among reproductive-age women in 10 SSA countries

			Hypertension status			
			Non-hypertensive		Hypertensive	
Variable	Year of survey	Weighted sample	%	n	%	n
Country			(χ^2^=2.1e+03, p<0.001)			
Benin	2017–2018	7522	90.05	6773	9.95	749
Burundi	2016–2017	16 725	99.00	16 558	1.00	168
Cameroon	2018	12 809	93.63	11 993	6.37	816
Ghana	2014	9098	92.45	8411	7.55	687
Gambia	2019	5922	85.68	5073	14.32	848
Kenya	2014	14 114	90.63	12 792	9.37	1322
Lesotho	2014	4197	82.43	3460	17.57	737
Madagascar	2021	9441	88.85	8388	11.15	1052
Mauritania	2019–2021	4823	85.81	4138	14.19	684
Zambia	2018	13 291	91.22	12 123	8.78	1168
All countries		97 942	91.60	89 711	8.40	8231

### Background characteristics by hypertension status

Table 2 indicates the distribution of hypertension status by the type of cooking fuel and the other background characteristics of respondents in the 10 SSA countries. Among respondents who used clean cooking fuel, 12.45% were hypertensive, compared with 7.80% among those with unclean cooking fuel. Hypertension was prevalent among older women (45–49 y) compared with younger ones (15–19 y). Regarding the level of education, the prevalence of hypertension was fairly evenly distributed among the various levels, ranging from 7.25% (no education) to 9.23% (secondary and higher). Approximately 3% of never-married women were hypertensive compared with 11% among formerly married women. Similarly, 5.16% of women with the poorest wealth index were hypertensive compared with 11.69% among those with the richest wealth index. Relatedly, respondents residing in urban area (11.02%), who did not visit a health facility in the last 12 months (9.84%), worked as a professional/managerial/clerical (12.70%), read a newspaper/magazine almost every day (14.39%), listened to radio almost every day (12.75%) and watched television almost every day (12.00%) were hypertensive compared with those residing in the rural area (6.64%), who visited a health facility in the last 12 months (6.41%), were not working (6.92%), did not read a newspaper/magazine at all (7.78%), did not listen to radio at all (5.82%) and did not watch television at all (6.19%). Using the χ^2^ test score, all independent variables selected for the study showed a significant association with hypertension.

**Table 2.** Background characteristics of respondents by hypertension status among reproductive-age women

	Hypertension status			
	Non-hypertensive		Hypertensive	
Explanatory variables	%	(n)	%	(n)
Type of cooking fuel				
Ungrouped	(χ^2^=705.0600, p<0.001)			
Electricity	85.11	2019	14.89	353
LPG	88.68	7113	11.32	908
Natural gas	87.28	1756	12.72	256
Biogas	79.21	258	20.79	68
Kerosene	86.90	1473	13.10	222
Coal, lignite	90.19	210	9.81	23
Charcoal	89.35	20 302	10.65	2421
Wood	93.41	53 797	6.59	3793
Straw/shrubs/grass	95.41	2230	4.59	107
Agricultural crops	89.46	74	10.54	9
Animal dung	86.16	270	13.84	43
Other	88.21	208	11.79	28
Grouped	(χ^2^=249.5170, p<0.001)			
Clean	87.55	11 166	12.45	1587
Unclean	92.20	78 545	7.80	6644
Age (years)	(χ^2^=2.7e+03, p<0.001)			
15–19	97.56	19 051	2.44	477
20–24	94.63	16 988	5.37	964
25–29	92.59	16 214	7.41	1298
30–34	90.21	12 905	9.79	1400
35–39	88.29	10 670	11.71	1416
40–44	85.18	7858	14.82	1367
45–49	82.16	6025	17.84	1308
Education	(χ^2^=37.8699, p<0.001)			
None	92.75	20 848	7.25	1629
Basic	91.82	31 858	8.18	2839
Secondary and above	90.77	37 005	9.23	3763
Marital status	(χ^2^=1.3e+03, p<0.001)			
Never married	96.65	27 010	3.35	937
Married	89.76	54 426	10.24	6208
Formerly married	88.40	8275	11.60	1086
Wealth index	(χ^2^=567.5839, p<0.001)			
Poorest	94.84	16 143	5.16	878
Poorer	93.40	16 742	6.60	1183
Middle	92.51	17 717	7.49	1434
Richer	90.19	18 691	9.81	2032
Richest	88.31	20 417	11.69	2703
Place of residence	(χ^2^=447.8823, p<0.001)			
Urban	88.98	35 087	11.02	4343
Rural	93.36	54 624	6.64	3887
Visited a health facility in the last 12 months	(χ^2^=359.9720, p<0.001)			
Yes	93.59	38 331	6.41	2623
No	90.16	51 380	9.84	5607
Occupation	(χ^2^=1.2e+03, p<0.001)			
Not working	93.08	29 398	6.92	2186
Professional/managerial/clerical	87.07	4070	12.93	604
Sales/service	87.30	16 929	12.70	2462
Agriculture	94.78	28 576	5.22	1574
Other	88.44	10 737	11.56	1404
Frequency of reading a newspaper/magazine	(χ^2^=179.1148, p<0.001)			
Not at all	92.22	73 269	7.78	6182
Less than once a week	89.22	9.787	10.78	1182
At least once a week	88.63	6336	11.37	812
Almost every day	85.61	319	14.39	54
Frequency of listening to radio	(χ^2^=526.4673, p<0.001)			
Not at all	94.18	37 175	5.82	2295
Less than once a week	90.98	18 836	9.02	1868
At least once a week	89.36	31 617	10.64	3763
Almost every day	87.25		12.75	304
Frequency of watching television	(χ^2^=694.1441, p<0.001)			
Not at all	93.81	50 803	6.19	3351
Less than once a week	89.56	11 690	10.44	1363
At least once a week	88.64	23 731	11.36	3041
Almost every day	88.00	3488	12.00	476

### Influence of the type of cooking fuel and risk of hypertension

Table 3 presents the results for all three models fitted for this study. The model fitness was assessed using the Prob>χ^2^ test, which indicated a highly significant result (p<0.001) for all three models, suggesting that the models fit the data well. The Akaike information criterion (AIC) was used for model comparison, with lower values indicating a better fit. The AIC values decreased from model 1 (54385.23) to model 3 (48129.02), suggesting an improvement in model fit. The pseudo-R^2^ values were also calculated to estimate the proportion of variance explained by the models. The pseudo-R^2^ increased from model 1 (0.0041) to model 3 (0.1201), indicating an increase in the explanatory power of the models in predicting hypertension risk. Our study showed that the type of cooking fuel, age, level of education, marital status, wealth index, visited a health facility in the last 12 months, occupation, exposure to the media (newspaper/magazine, radio and television) and country of residence had a statistically significant association with the risk of hypertension.

**Table 3.** Bivariate and multivariate analysis of factors associated with the risk of hypertension

	Model 1,	Model 2,	Model 3,
Explanatory variables	OR (95% CI)	AOR (95% CI)	AOR (95% CI)
Type of cooking fuel			
Clean	Ref	Ref	Ref
Unclean	0.6*** (0.56 to 0.64)	0.9 (0.89 to 1.03)	1.21*** (1.11 to 1.31)
Age (years)			
15–19		Ref	Ref
20–24		1.61*** (1.42 to 1.83)	1.60*** (1.41 to 1.18)
25–29		1.93*** (1.69 to 2.20)	1.95*** (1.71 to 2.22)
30–34		2.41*** (2.10 to 2.76)	2.45*** (2.14 to 2.80)
35–39		3.16*** (2.76 to 3.62)	3.18*** (2.78 to 3.64)
40–44		4.28*** (3.73 to 4.91)	4.27*** (3.73 to 4.89)
45–49		5.80*** (5.05 to 6.66)	5.78*** (5.04 to 6.62)
Education			
None		Ref	Ref
Basic		2.01*** (1.06 to 1.22)	1.12** (1.04 to 1.21)
Secondary and above		1.94*** (1.73 to 2.18)	1.14** (1.05 to 1.23)
Marital status			
Never married		Ref	Ref
Married		2.01*** (1.82 to 2.21)	1.64*** (1.49 to 1.80)
Formerly married		1.94*** (1.73 to 2.18)	1.69*** (1.51 to 1.90)
Wealth index			
Poorest		Ref	Ref
Poorer		1.18*** (1.08 to 1.29)	1.24*** (1.13 to 1.35)
Middle		1.17** (1.07 to 1.27)	1.32*** (1.21 to 1.45)
Richer		1.26*** (1.15 to 1.38)	1.63*** (1.48 to 1.80)
Richest		1.26*** (1.14 to 1.40)	1.97*** (1.75 to 2.21)
Place of residence			
Urban		Ref	Ref
Rural		0.94** (0.85 to 0.97)	0.94 (0.88 to 1.00)
Visited a health facility in the last 12 months			
Yes		Ref	Ref
No		1.40*** (1.33 to 1.48)	1.59*** (1.51 to 1.68)
Occupation			
Not working		Ref	Ref
Professional/managerial/clerical		0.94 (0.84 to 1.05)	1.15** (1.03 to 1.29)
Sales/service		1.19*** (1.11 to 1.28)	1.34*** (1.24 to 1.44)
Agriculture		0.63*** (0.58 to 0.67)	1.01 (0.93 to 1.09)
Other		1.12** (1.04 to 1.21)	1.20*** (1.11 to 1.31)
Frequency of reading a newspaper/magazine			
Not at all		Ref	Ref
Less than once a week		1.16*** (1.07 to 1.25)	1.09* (1.01 to 1.18)
At least once a week		1.11** (1.01 to 1.22)	1.06 (0.96 to 1.16)
Almost every day		1.27 (0.90 to 1.79)	1.21 (0.86 to 1.71)
Frequency of listening to radio			
Not at all		Ref	Ref
Less than once a week		1.22*** (1.14 to 1.31)	1.45*** (1.07 to 1.23)
At least once a week		1.32*** (1.24 to 1.41)	1.30*** (1.22 to 1.39)
Almost every day		1.42*** (1.22 to 1.66)	1.23*** (1.22 to 1.66)
Frequency of watching television			
Not at all		Ref	Ref
Less than once a week		1.33*** (1.23 to 1.44)	1.09* (1.00 to 1.17)
At least once a week		1.27*** (1.18 to 1.36)	1.02 (0.94 to 1.10)
Almost every day		1.41*** (1.23 to 1.62)	1.23** (1.05 to 1.43)
Country			
Benin			Ref
Burundi			0.11*** (0.90 to 0.13)
Cameroon			0.69*** (0.61 to 0.77)
Ghana			0.59*** (0.52 to 0.66)
Gambia			1.40*** (1.25 to 1.57)
Kenya			0.72*** (0.65 to 0.80)
Lesotho			1.38*** (1.21 to 1.57)
Madagascar			1.11 (0.99 to 1.23)
Mauritania			1.59*** (1.39 to 1.81)
Zambia			0.74*** (0.66 to 0.84)
Constant	0.000	0.000	0.000
Model fitness			
Prob>χ^2^	<0.001	<0.001	<0.001
AIC	54385.23	50009.58	48129.02
Pseudo-R^2^	0.0041	0.0853	0.1201
N	97 942	97 942	97 942

Ref: reference category. *p<0.05, **p<0.01, ***p<0.001.

In model 1, we used a bivariate analysis to assess the direct influence of the type of cooking fuel on the risk of hypertension. The results demonstrate that women using unclean cooking fuel were less likely to be hypertensive (OR 0.6 [95% CI 0.56 to 0.64]) compared with those who were using clean cooking fuel. However, this direction of association shifted, showing a positive association between unclean cooking fuel and the risk of hypertension after adjusting for some identified demographic and socio-economic factors together with country of residence in the final model (model 3). Thus, compared with women who use clean cooking fuel, those with unclean cooking fuel were 1.21 times more likely to be hypertensive (AOR 1.21 [95% CI 1.11 to 1.31]).

Relatedly, the final model showed an increased risk of hypertension with increasing age. The likelihood of being hypertensive was 5.78 times greater among older women (AOR 5.78 [95% CI 5.04 to 6.62]) compared with younger women. A higher likelihood of being hypertensive was observed among respondents with a basic education (AOR 1.12 [95% CI 1.04 to 1.21]) and secondary and above (AOR 1.14 [95% CI 1.05 to 1.23]) than those with no education. Women who were married (AOR 1.64 [95% CI 1.49 to 1.80]) or formerly married (AOR 1.69 [95% CI 1.51 to 1.90]) had higher odds of being hypertensive than never-married women. We observed an increased risk of hypertension with an increase in wealth index category. For instance, respondents in the richest wealth index were 1.97 times more likely to be hypertensive than those in the poorest wealth index. A similar association was observed among the other wealth categories compared with the reference category (poorest). Respondents who visited a health facility in the last 12 months had higher odds of being hypertensive (AOR 1.59 [95% CI 1.51 to 1.68]) than their counterparts who did not.

Furthermore, we found a positive association between the type of occupation and hypertension. Thus respondents within the professional/clerical/managerial (AOR 1.15 [95% CI 1.03 to 1.29]) and sales/service (AOR 1.34 [95% CI 1.24 to 1.44]) occupations had higher odds of being hypertensive than those who were not working. The study demonstrated a significant association between exposure to the media and the risk of hypertension. Respondents who listen to the radio (AOR 1.23 [95% CI 1.22 to 1.66]) or who watch television (AOR 1.23 [95% CI 1.05 to 1.43]) almost every day had a higher likelihood of being hypertensive compared with those who did not listen to the radio or did not watch television at all.

Finally, we found disparities in the risk of hypertension among the sampled countries. Women from Gambia (AOR 1.40 [95% CI 1.25 to 1.57]), Lesotho (AOR 1.38 [95% CI 1.21 to 1.57]) and Mauritania (AOR 1.59 [95% CI 1.39 to 1.81]) had higher odds of being hypertensive compared with the reference country (Benin). However, compared with the reference country, women in Burundi, Cameroon, Ghana, Kenya and Zambia were less likely to be hypertensive.

## Discussion

In this study we sought to assess the association between the type of cooking fuel and hypertension risk among women in SSA. Consistent with some previous studies,^12,13^ we found a significant association. In our initial analyses, the direction of the association was inverse, i.e. unclean cooking fuel use was associated with a lower risk of hypertension. However, after adjusting for covariates, there was a directional change, with the risk of hypertension being significantly higher among those who used unclean cooking fuel. The positive association can be explained from the perspective that the combustion of unclean cooking fuels produces high levels of indoor air pollution, including fine particulate matter (<2.5 μm), carbon monoxide and nitrogen oxides.^30,31^ Exposure to these pollutants has been linked to an increased risk of hypertension by promoting oxidative stress, inflammation and endothelial dysfunction.^32,33^ Therefore, our findings underscore a need for SSA countries to intensify current actions to reduce the reliance on unclean cooking fuel at the household and population level.

Our analysis of the covariates confirmed previous studies^4,24,25^ that found the risk of hypertension to be significantly associated with age, level of education, marital status, type of occupation, wealth index, exposure to the media and frequency of visiting a health facility. Older women were 5.78 times more likely to be hypertensive compared with younger women. Biologically, ageing is characterised by some systemic alterations in the endothelium morphology, thereby resulting in decreased endothelial function.^34,35^ This pathway is a known risk factor for hypertension, especially when older people are additionally exposed to indoor air pollution.^35^

Our results show that a higher wealth index is associated with a higher risk of hypertension. Similar findings have been reported in studies conducted in Ghana^27^ and South Asia.^26^ Nyarko^27^ posits that the association between wealth and the risk of hypertension is explained by the fact that individuals from wealthy households tend to live a sedentary life devoid of substantial physical activity (a known protective factor against hypertension and other chronic conditions). Therefore it is not surprising to see the risk of hypertension significantly higher among women working in professional/clerical/managerial and sales and service occupations. Such occupations are mostly sedentary in nature, thereby posing as a double burden.

Consistent with previous literature,^27,36^ the study found the likelihood of being hypertensive to be significantly higher among women with higher educational attainment. This observation can be explained by the fact that higher education increases the chances of women gaining employment and receiving a high income, which can then increase their accessibility to unhealthy foods, thus increasing the risk of hypertension. The WHO^1^ states that 46% of persons with hypertension are unaware of their status. Our findings regarding education and hypertension risk suggest women who have high educational attainment are more likely to be screened and know their status.

The study also revealed a significant positive association between media exposure and hypertension risk, a result that mirrors previous studies.^29^ For instance, the result is congruent with the findings of Kim et al.,^29^ who found a high frequency of watching TV to be associated with a higher risk of hypertension. As watching TV is a sedentary activity, this explains the observed association. Another perspective could be that exposure to the media equips women with health information that encourages them to check their blood pressure, thereby making them more likely to know their status.

Compared with women who visited a health facility within the last 12 months, those who did not visit a health facility had a 13% greater likelihood of being hypertensive. The findings align with a previous study conducted in Nigeria^28^ that reported significantly higher odds of hypertension among individuals who did not visit a health facility. One possibility is that women who did not visit a health facility may have less knowledge about hypertension and its risk factors, which could lead to unhealthy behaviours that increase their risk of developing hypertension. Consistent with a study conducted in Ghana,^27^ we found the odds of hypertension to be significantly high among those currently married or previously married. Further studies are required to fully understand the association between marital status and hypertension risk.

### Strengths and limitations

A major limitation of this study is that we were unable to establish causality between the type of household cooking fuel and the risk of hypertension. Also, due to the nature of the study, we were unable to determine whether the outcome (being hypertensive) occurred before using the current type of household cooking fuel. Similarly, there is a possibility that outdoor air pollution might interfere with the studied association; however, the DHS dataset does not provide information on outdoor air pollution. The self-reported nature of the data also exposes the study to recall bias. Nonetheless, the large nationally representative dataset used supports the generalisability of our findings. Also, we used appropriate analytical tools to arrive at our findings.

## Conclusions

In conclusion, there is a need for SSA countries to intensify efforts to reduce the reliance on unclean cooking fuel at the household and population levels. This could be achieved through promoting the use of clean cooking technologies and fuels such as LPG, biogas or electricity, as well as implementing policies that support the transition from unclean cooking fuels. Interventions aimed at reducing hypertension risk among women in SSA must be tailored to target those who use unclean cooking fuel, older women, those in higher-wealth households and those with higher education. Encouraging health facility visitation can be a conduit for educating women on hypertension and its risk factors.

## Data Availability

The dataset is freely accessible for download at https://dhsprogram.com/data/available-datasets.cfm.
